# Inactivated rabies-vectored SARS-CoV-2 vaccine provides long-term immune response unaffected by vector immunity

**DOI:** 10.1038/s41541-022-00532-7

**Published:** 2022-09-23

**Authors:** Catherine Yankowski, Christoph Wirblich, Drishya Kurup, Matthias J. Schnell

**Affiliations:** 1 Department of Microbiology and Immunology, Sidney Kimmel Medical College at Thomas Jefferson University, Philadelphia, PA USA; 2 Jefferson Vaccine Center, Sidney Kimmel Medical College, Thomas Jefferson University, Philadelphia, PA USA

**Keywords:** Inactivated vaccines, Antibodies, SARS-CoV-2, Adjuvants

## Abstract

The objective of this study is to further analyze recombinant rabies virus-vectored SARS-CoV-2 vaccine, CORAVAX, as an effective COVID-19 vaccine strategy. CORAVAX has proven immunogenic and protective against SARS-CoV-2 in animal models. Here, we have screened adjuvants for the highest quality antibody titers, negated the concern of pre-existing rabies-vector immunity, and established its potential as a long-term COVID-19 vaccine. We have tested toll-like receptor 4 (TLR4) agonists, inflammasome activators, and alum adjuvants in CORAVAX and found TLR4-activating MPLA-AddaVax to have the greatest potential. We followed the humoral immune response to CORAVAX in mice with pre-existing rabies virus immunity and saw no significant differences compared to naive mice. We then followed the immune response to CORAVAX over several months and 1-year post-immunization. Mice maintained high antigen-specific serum antibody titers as well as long-lived antibody-secreting cells in the spleen and bone marrow. We believe this rabies-vector strategy combats the problem of waning immunity of other COVID-19 vaccines. These results together support CORAVAX’s potential during the ongoing COVID-19 pandemic.

## Introduction

Severe acute respiratory syndrome coronavirus 2 (SARS-CoV-2) emerged in December 2019 in Wuhan, China, and rapidly engulfed the world in a coronavirus disease (COVID-19) pandemic. More than 514 million people have been infected, resulting in over 6 million deaths^1^. Despite two-thirds of the US population being fully vaccinated, as of May 2022, there are still over 2300 deaths weekly in the USA (Johns Hopkins University)^1^. There are currently 195 preclinical COVID-19 vaccines with 3 reaching FDA approval (World Health Organization)^2^. Our rabies virus (RABV)-vectored COVID-19 vaccine containing the S1 domain of the SARS-CoV-2 spike glycoprotein, CORAVAX, has proven immunogenic in mice and protective in hamsters^3,4^.

Inactivated RABV vaccines have been extensively studied, widely used, and have a long history of safe administration in humans^5^. Our research has expansively studied recombinant RABV vaccines against emerging pathogens for over a decade^6–10^. The RABV genome allows for insertion of the foreign gene of interest for expression and incorporation into virions. A stable mutation at the amino acid position 333 of the RABV glycoprotein attenuates the virus and abolishes its neurovirulence^11^. The administration of inactivated viral particles makes the vaccine safe for immunocompromised individuals and pregnant women. Developed for multiple pathogens, this inactivated RABV platform has proven protective in mice and non-human primates (for a comprehensive list see cited review)^12^.

The ability of adjuvants to affect vaccine immune responses is an important consideration in vaccine development, especially for inactivated vaccine platforms. We have established the efficacy of toll-like receptor 4 (TLR4) agonists, particularly glucopyranosyl lipid adjuvant in a stable oil-in-water emulsion (GLA-SE) in the recombinant RABV platform^6,8,13^. Due to its commercial unavailability, we switched to another, similar molecule with available components MPLA-AddaVax (Invivogen). Both of these adjuvants significantly increase antibody titers and skew the immune system toward a Th1, antiviral response. As a control, we included the gold standard adjuvant used for decades, alum, the aluminum salts used in the majority of FDA-approved vaccines.

Every year, >29 million people worldwide receive a post-bite vaccination for RABV^14^. Many people who are at high risk for exposure receive preventative vaccination, including laboratory workers, veterinarians, animal handlers, spelunkers, and travelers going to parts of the world where exposure to RABV is likely^15^. However, we wanted to ensure that prior RABV immunity has no impact on the immunogenicity of CORAVAX. A RABV-based vaccine for COVID-19 has the advantage of also protecting vaccinees from RABV, as the virus is still responsible for an estimated 59,000 global human deaths annually^16,17^. Endemic regions coincide with the developing world, still in dire need of distribution of an effective COVID-19 vaccine. Thus, we sought to investigate whether prior vaccination for RABV would impact the ability of CORAVAX to rapidly increase S1-specific antibody titers.

Despite the efficacy of current vaccines, coronavirus immunity has been shown to wane rapidly^18^. The mRNA platforms for the BioNTech, Pfizer COVID-19 vaccine (BNT162b2) and Moderna, NIAID COVID-19 vaccine (mRNA-1273) demonstrate efficacy, but a fourth inoculation is now recommended in <2 years for individuals over 50 years old in the United States. Another lead candidate, the Janssen Pharmaceuticals adenovirus-based vaccine (JNJ-78436735J&J), showed evidence of waning protection with breakthrough infections as early as 4 months^19^. Failure to control SARS-CoV-2 spread is leading to consistently emerging variants of concern and hindering vaccination efforts. Vaccination with the RABV vaccine often protects long-term and revaccination is rarely needed even after decades of previous immunization. Due to the severity of rabies disease, vaccination of at-risk individuals is recommended every 10 years; however, 97% of immunocompetent individuals demonstrate protective levels of neutralizing antibodies at 10 years^20^.

Thus, we investigated two adjuvants, the effects of pre-existing RABV-vector immunity, and the long-term immunity of CORAVAX for its potential as an effective, long-lasting SARS-CoV-2 vaccine. To confirm that the RABV-based vaccine has the potential to be protective in the long term, we followed the immune response to CORAVAX in mice for 1 year to identify antibody-secreting cells (ACSs) contributing to highly maintained serum antibody titers.

## Results

### MPLA-AddaVax adjuvanted CORAVAX elicits high S1-specific antibody titers with a Th1/Th2 balance

**Fig. 1:**
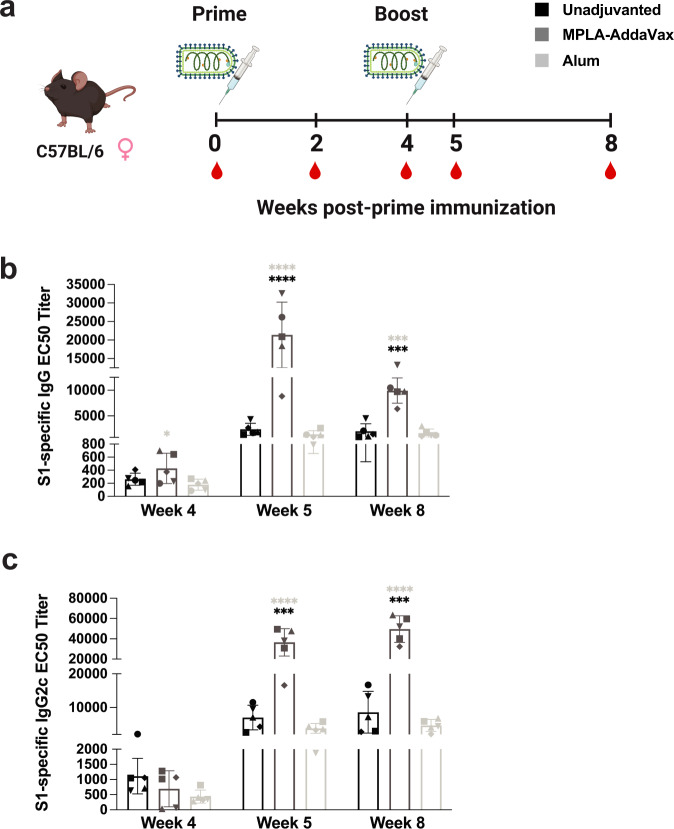
Adjuvant comparison in RABV-vectored SARS-CoV-2 vaccine CORAVAX. **a** Schematic of the immunization schedule in C57BL/6 mice (*n* = 5 per group, female) with prime and boost of indicated adjuvant groups. Mice were immunized with 10 µg of CORAVAX without adjuvant, with MLPA-AddaVax, or with alum at day 0 and week 4. Sera were collected at 2, 4, 5, and 8 weeks post-immunization and analyzed by ELISA. **b** S1-specific IgG ELISA at weeks 4, 5, and 8 post-prime immunization was reported as average half-maximal effective concentration (EC50) titer (bars) determined from individual mouse serum (symbols) ELISA curves. **c** Average EC50 titers (bars) determined from individual mouse (symbols) ELISA curves of anti-S1 serum IgG2c antibodies at weeks 4, 5, and 8. Error bars represent the standard deviation (SD) from the mean. Statistics are by one-way ANOVA with post-hoc Tukey’s test of log-transformed EC50 titers. *p* > 0.1234 (ns), *p* < 0.0332 (*), *p* < 0.0021 (**), *p* < 0.0002 (***), *p* < 0.0001 (****).

For the preclinical development of CORAVAX, an adjuvant comparison was completed looking at total S1-specific antibody titers and isotype subclass responses. C57BL/6 mice were immunized with CORAVAX without adjuvant, with MPLA-AddaVax, or with alum (Fig. 1a). In our experiments, a minimum of 5 mice per group were used to obtain statistical power. On the day of the boost immunization at week 4, the TLR4 agonist adjuvant MPLA-AddaVax had the highest S1 IgG titers after a single dose. After the boost immunization, MPLA-AddaVax showed a nearly ninefold increase in titers compared to unadjuvanted controls, and this response continued one-month post-boost at week 8. Alum-adjuvanted mice did not see an increase in IgG titers above unadjuvanted controls (Fig. 1b). Looking at the isotype subclass responses in C57BL/6 mice wherein IgG2c is the Th1-associated antibody, minor differences were seen between adjuvant groups at week 4. However, after the boost at weeks 5 and 8, the IgG2c response was significantly highest for MPLA-AddaVax (Fig. 1c). Of note, alum-adjuvanted mice had even lower IgG2c titers than unadjuvanted. In terms of IgG1 responses, MPLA-AddaVax had the highest responses overall (Supplementary Fig. 1). Due to MPLA-AddaVax having robust responses for both IgG2c and IgG1, subsequent studies moved forward with the CORAVAX and MPLA-AddaVax formulation.

### Pre-existing RABV immunity has no impact on the antibody response to CORAVAX

**Fig. 2:**
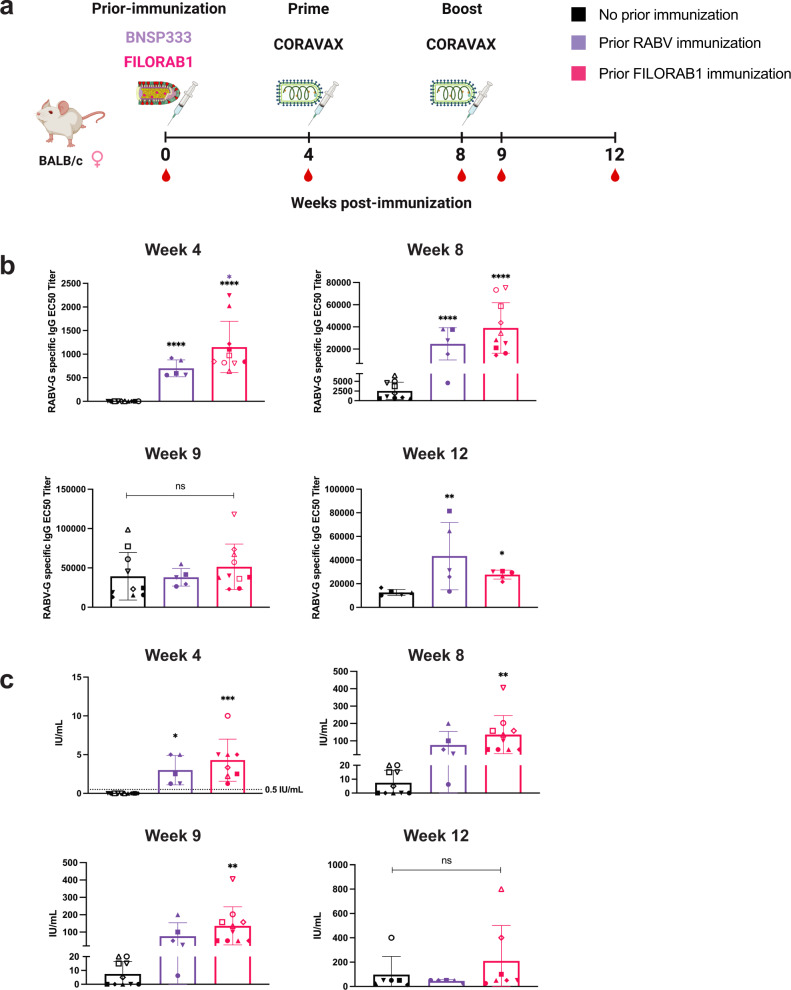
Establishing RABV immunity prior to CORAVAX immunization. **a** Schematic of immunization in BALB/c mice (*n* = 5 per group, female) with a RABV-naive control group, a RABV vaccine BNSP333 immunized group, and a RABV-vectored EBOV vaccine immunized group. CORAVAX immunization followed afterward by prime-boost schedule at weeks 4 and 8. Sera were collected at weeks 4, 8, 9, and 12. **b** RABV-G-specific IgG ELISA at week 4 pre-CORAVAX immunization and weeks 8–12 both before and after the CORAVAX boost. Average EC50 titers (bars) are determined from individual mouse (symbols) ELISA curves. Individual mice from a pilot experiment are included (open symbols). **c** RABV-neutralizing antibody titers by RFFIT reported in international units (IU) per mL. Average neutralizing titers (bars) of individual mouse serum (symbols) including a pilot experiment (open symbols). 0.5 IU/mL is considered the accepted protective level (dotted line). Error bars represent SD from the mean. Statistics are by one-way ANOVA with post-hoc Tukey’s test of log-transformed EC50 or neutralizing titers. *p* > 0.1234 (ns), *p* < 0.0332 (*), *p* < 0.0021 (**), *p* < 0.0002 (***), *p* < 0.0001 (****).

To test the impact of prior RABV immunization on the immune response to a RABV-vectored vaccine, two groups of BALB/c mice were immunized with recombinant RABV vaccines, either BNSP333 (the empty rabies vector expressing only native RABV-G), or FILORAB1 (the RABV-based Ebola virus (EBOV) vaccine expressing RABV-G as well as EBOV-GP). A 3rd group received no prior RABV immunization. All 3 groups were then immunized with CORAVAX following the prime-boost regimen (Fig. 2a).

RABV-immunized groups demonstrated seroconversion at week 4 by RABV-G-specific IgG ELISA, while the control group did not (Fig. 2b). RABV-immunized groups also had neutralizing antibody titers above the accepted protective level of 0.5 IU/mL (Fig. 2c). All mice in which prior RABV immunity was established were therefore considered RABV immune in this study. Following a single dose of CORAVAX, RABV-immunized groups exhibit a boosted response, but by week 9 all three groups had similar levels of RABV-G IgG and neutralizing titer. At the end of the study at week 12, RABV-immunized mice that responded to the antigen three times maintained significantly higher titers by ELISA, but had no significant difference in neutralization.

**Fig. 3:**
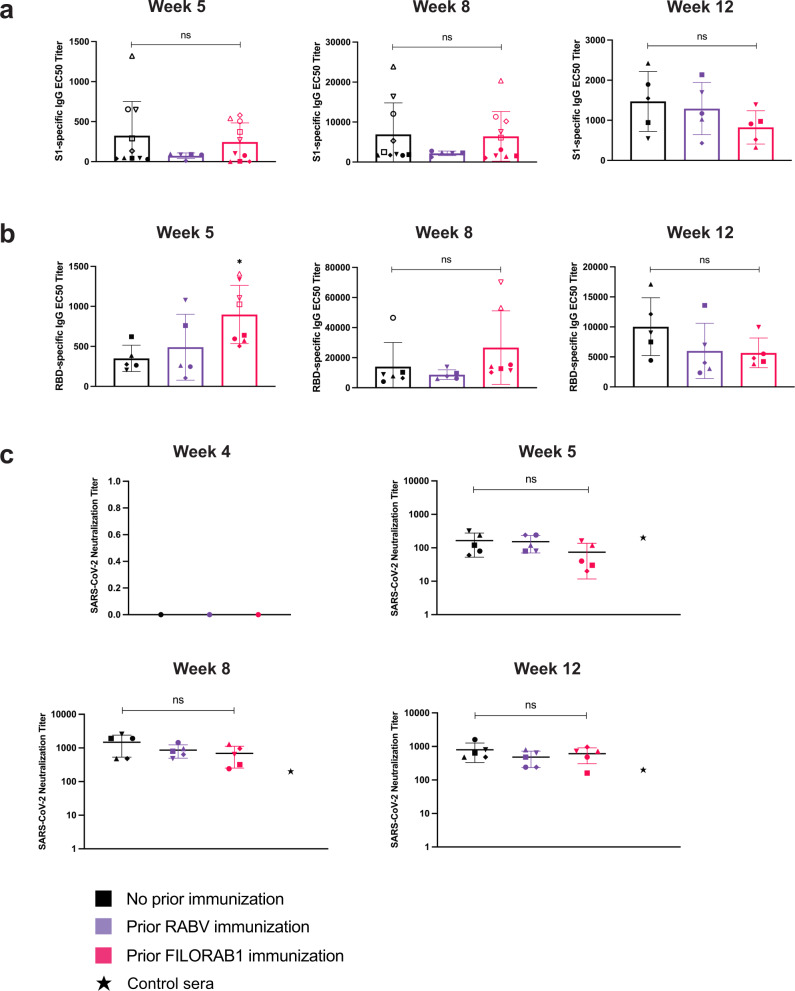
Effect of pre-existing RABV immunity on SARS-CoV-2 specific responses. **a** S1-specific and **b** RBD-specific IgG ELISA in CORAVAX-immunized mouse sera at weeks 8–12 following prior RABV immunity. The average IgG EC50 titers (bars) are determined from individual mouse (symbols) ELISA curves, including a pilot experiment (open symbols). **c** SARS-CoV-2 neutralization assay for log-transformed neutralizing antibody titers at week 4 before CORAVAX immunization and weeks 5–12 in CORAVAX-immunized mouse sera. The line represents the average neutralization titer per group of individual mice (symbols). The positive control serum (black star) is from a previous experiment of CORAVAX-immunized hamsters at week 8. Error bars represent SD from the mean. Statistics are by one-way ANOVA with post-hoc Tukey’s test of log-transformed EC50 or neutralizing titers. *p* > 0.1234 (ns), *p* < 0.0332 (*), *p* < 0.0021 (**), *p* < 0.0002 (***), *p* < 0.0001 (****).

The S1-specific responses demonstrated no significant differences throughout the experiment, independent of prior RABV immunity (Fig. 3a). Initially, after a single CORAVAX immunization, mice previously immunized with FILORAB1 had a higher RBD-specific IgG titer on week 5, but after the boost immunization, this was not significantly different on weeks 8 and 12 (Fig. 3b). Our SARS-CoV-2 neutralizing titers are reported as the dilution capable of 100% virus neutralization, and all groups had similar neutralizing capacities (Fig. 3c). Neutralizing titer was compared to positive control sera from week 8 of a two-dose hamster experiment (unpublished). Therefore, we believe from these results that there will likely be no issue of prior RABV immunity before CORAVAX administration in the human population. We can conclude that the RABV-vector strategy, as a inactivated vaccine, is not hindered by pre-existing immunity documented in other live viral-vectored vaccines.

### CORAVAX establishes long-term immune responses 1-year post-immunization in mice

**Fig. 4:**
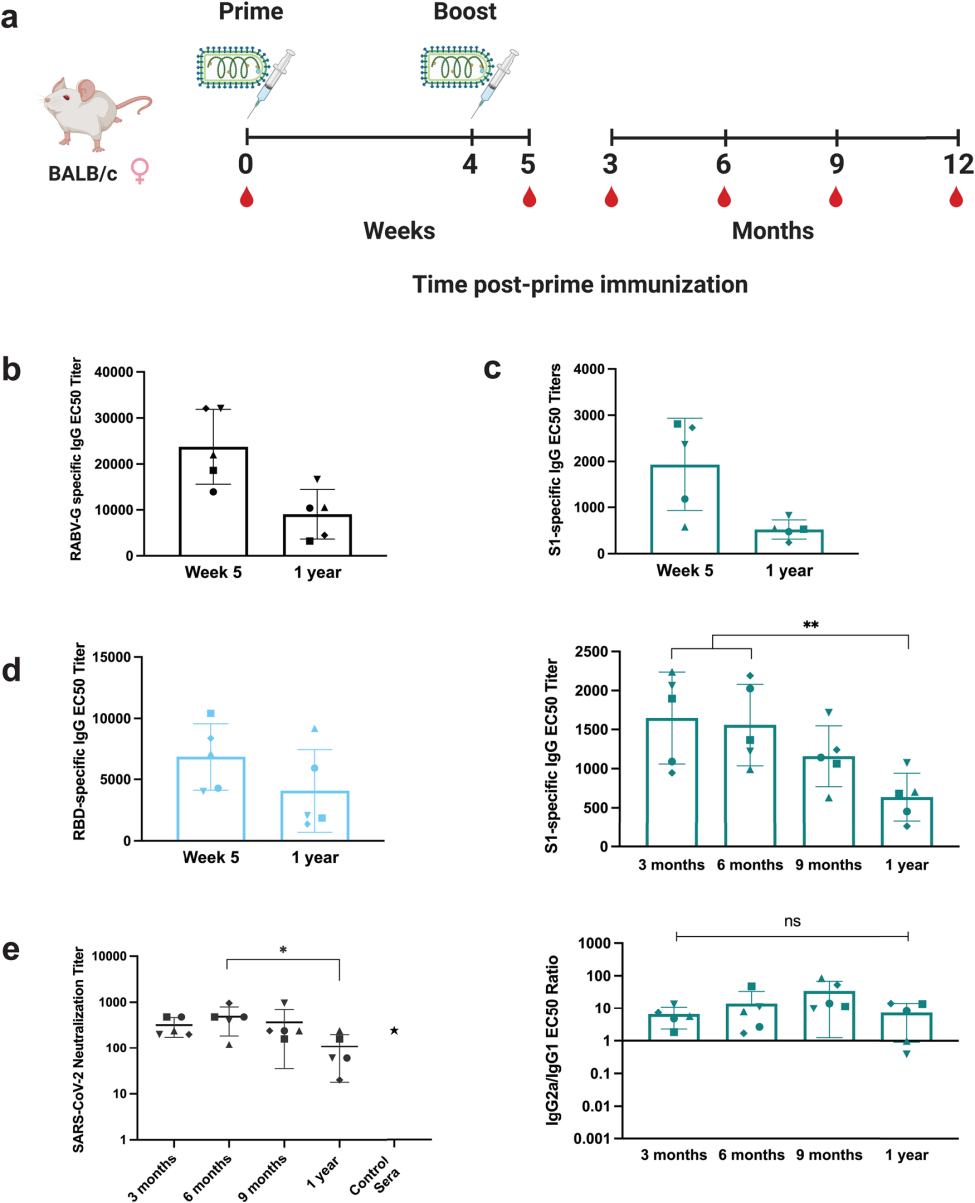
CORAVAX immunization results in long-term serum antibody titers in mice. **a** Immunization schedule for BALB/c mice (*n* = 5 per group, female) and endpoint of sera collection 3–12 months post-immunization. **b** RABV-G **c** S1 and **d** RBD-specific IgG ELISA of immunized mouse sera 1-week post-boost immunization (week 5) compared to 1 year. Average EC50 titers (bars) were determined from individual mouse (symbols) ELISA curves. **c** Additionally for S1-specific responses, the average ratio of IgG2a/IgG1 EC50 titers over time from 3 months to 1 year. **e** SARS-CoV-2 neutralization assay for log-transformed neutralizing antibody titers over time in CORAVAX-immunized mouse sera up to 1 year. The line represents the average neutralization titer per group of individual mice (symbols). The positive control serum (black star) is from a previous experiment of CORAVAX-immunized hamsters at week 8. Error bars represent SD from the mean. Statistics are by one-way ANOVA with post-hoc Tukey’s test of log-transformed EC50 or neutralizing titers. *p* > 0.1234 (ns), *p* < 0.0332 (*), *p* < 0.0021 (**), *p* < 0.0002 (***), *p* < 0.0001 (****).

BALB/c mice were immunized following the standard schedule, and each group was sacrificed at either 3, 6, 9, or 12 months post-immunization (Fig. 4a). For all time points, serum was analyzed for S1-specific antibodies by ELISA and SARS-CoV-2 neutralizing antibody titers; 1-year serum was also analyzed for antibody responses to RABV-G and the receptor-binding domain (RBD) of S1. Peak titers following CORAVAX immunization are seen 1-week post-boost immunization at week 5. All mice had similar S1-specific IgG titers at this time point (Supplementary Fig. 2). Antibody titers at each group’s endpoint were compared to the peak titers at week 5. RABV vaccination is known to elicit long-term immunity in the host. Comparing week 5 to 1-year post-immunization, we found maintenance of half of the RABV-G titer (Fig. 4b). This was also the case for the S1-specific antibody levels. Following the S1 response in these mice over several months, we found a gradual decrease of serum antibody titers, with subclass ratios remaining consistently skewed toward Th1 (Fig. 4c). S1-specific titers decreased significantly from 3 and 6 months to 1 year, but not between 9 months and 1 year, with titers well maintained over time. The same can be said for the RBD of S1 (Fig. 4d). For SARS-CoV-2-neutralizing antibody titers, we found peak neutralization at 6 months, followed by a gradual decline at 1 year (Fig. 4e). Similarly, 1-year neutralizing titers were not much lower than those of CORAVAX-immunized hamsters at week 8 (positive control) as found in a previous experiment in which these animals were protected from SARS-CoV-2 challenge (unpublished).

### Long-term serum titers are a result of long-lived ASCs residing in the spleen and bone marrow

**Fig. 5:**
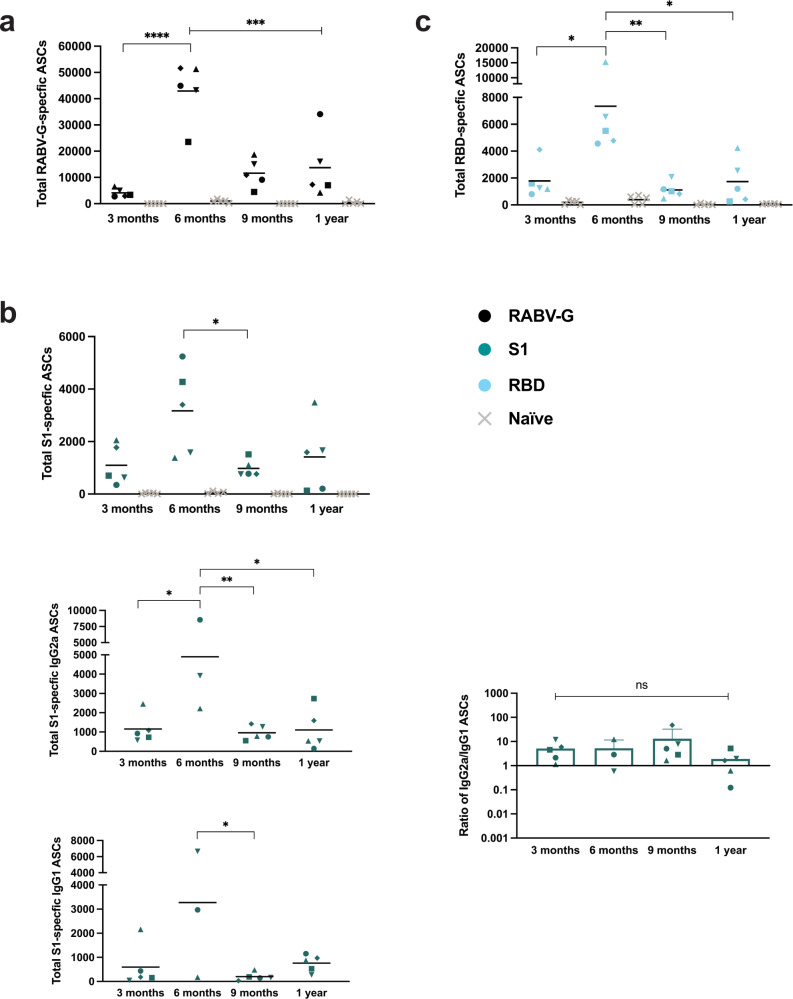
Detection of antigen-specific antibody-secreting cells (ASCs) in the spleens of CORAVAX-immunized mice. **a** The average total number (line) of RABV-G **b** S1 and **c** RBD-specific ASCs present in individual mouse spleens (symbols) at 3 months, 6 months, 9 months, and 1 year compared to naive controls (gray × symbols). **b** Additionally for S1, average total number of IgG2a and IgG1 ASCs (lines) from individual mice (symbols) and ratio of IgG2a/IgG1 ASCs. Statistics are by one-way ANOVA with post-hoc Tukey’s test of log-transformed total number of cells or ratios. *p* > 0.1234 (ns), *p* < 0.0332 (*), *p* < 0.0021 (**), *p* < 0.0002 (***), *p* < 0.0001 (****).

We assayed for the presence of antigen-specific antibody-secreting cells (ASCs) by ELISpot in the spleens of immunized mice. Compared to naive mice, the RABV-G-specific ASCs were present to the highest extent at 6 months post-immunization but were still abundantly present at 1 year (Fig. 5a). We discovered a robust number of S1-specific cells in the spleens of these mice as well (Fig. 5b). Looking at the specific isotype subclass that these cells were secreting, we found a larger number of IgG2a-specific cells than IgG1. Although the ratio of IgG2a to IgG1 decreases at 1 year, these ratios were not significantly different. For RBD-specific ASCs, we found the peak number at 6 months, but still over 1000 cells per spleen on average at 1 year (Fig. 5c).

**Fig. 6:**
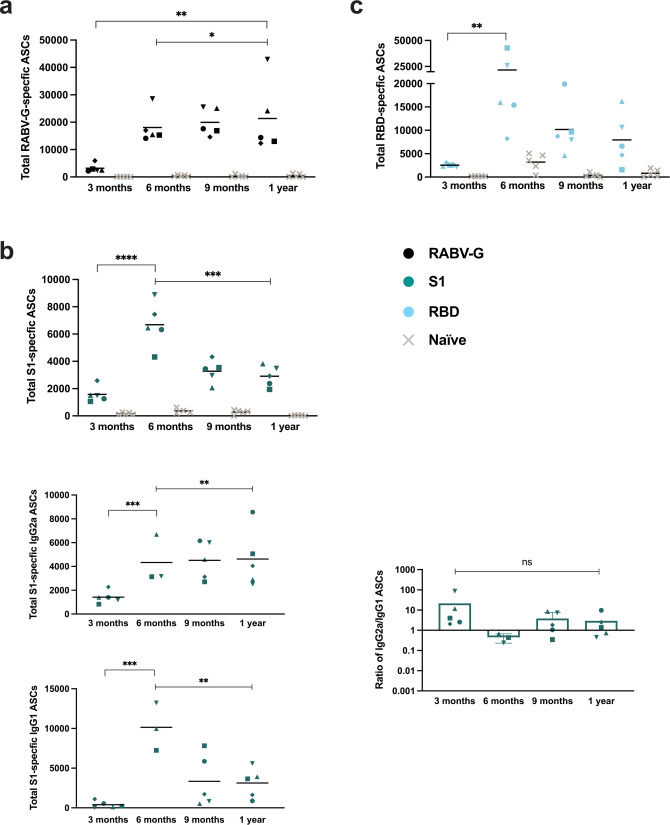
Long-term responses to CORAVAX are maintained by long-lived antibody-secreting cells (ASCs) in the bone marrow. **a** The average total number (line) of RABV-G **b** S1 and **c** RBD-specific ASCs present in individual mouse bone marrow (symbols) at 3 months, 6 months, 9 months, and 1 year compared to naive controls (gray × symbols). The line represents the average per group. **b** Additionally for S1, average total number of IgG2a and IgG1 ASCs (lines) from individual mice (symbols) and ratio of IgG2a/IgG1 ASCs. Statistics are by one-way ANOVA with post-hoc Tukey’s test of the log-transformed total number of cells or ratios. *p* > 0.1234 (ns), *p* < 0.0332 (*), *p* < 0.0021 (**), *p* < 0.0002 (***), *p* < 0.0001 (****).

In the bone marrow, we expected to see an even more long-lived signature, as is typical of ASCs residing in this location. On average, RABV-G-specific ASCs were more highly maintained for 1 year (Fig. 6a). We also saw S1-specific cells in the bone marrow following the same pattern as in the spleen (Fig. 6b). As was the case in the spleen, we see a greater skew toward Th1-associated cells with ratios slightly decreasing at 1 year, but not significantly different from earlier time points. Lastly, for RBD-specific ASCs, we found a much larger number of cells in the bone marrow than in the spleen, with the peak number also at 6 months (Fig. 6c). We believe these cells in the spleen and bone marrow are the main contributor to serum antibody levels in these long-term studies. Taken together, the results of our long-term mouse experiments highlight the remarkable longevity of immune responses to the RABV-vector platform and add much evidence to suggest CORAVAX’s potential as an effective COVID-19 vaccine in the human population.

## Discussion

We have demonstrated that the RABV-based SARS-CoV-2 vaccine CORAVAX adjuvanted with MPLA-AddaVax elicits high serum antibody titers against S1, with no effects of pre-existing RABV immunity, and that these titers are robustly maintained at 1 year post-immunization. The RABV-vector strategy has been extensively studied and widely used. The vector’s attenuating mutation is stable and an additional safety measure for vaccine production, but only an inactivated RABV-vectored vaccine would be considered for human use. The human RABV vaccine is an inactivated platform that has been safe and effective for over 50 years. CORAVAX has been tested in a phase I human clinical trial in India by Bharat Biotech International Ltd.^21^, and our RABV-vectored Lassa fever vaccine is entering a phase I clinical trial in the US.

Following this study, CORAVAX adjuvanted with MPLA-AddaVax has proven protective from SARS-CoV-2 challenge in hamsters^4^. Compared to control hamsters immunized with the rabies-based EBOV vaccine FILORAB1, CORAVAX-immunized hamsters had minimal inflammation and viral presence in the lungs and nasal mucosa. We have not tested whether the TLR4 agonist adjuvant is required for protection, but it has been shown in our previous study to increase immunogenicity and neutralizing titers^3^. Adjuvants are useful components of modern vaccines for rapid responses, dose sparing, reducing the number of immunizations, and overcoming immune senescence in the elderly^22^. All of these are reasons we include an adjuvant in the CORAVAX immunization. However, we speculate that it is the role of the RABV vector, and not the adjuvant, that increases the longevity of the immune response. Our evidence includes a study of FILORAB1 in which robust antibody titers were maintained in mice for 1 year, but with no difference between unadjuvanted and various adjuvant groups, including TLR4 agonists (unpublished).

A branch of COVID-19 candidate vaccines, including the Janssen Pharmaceuticals JNJ-78436735, utilize adenovirus vectors, where pre-existing vector immunity can be a problem. The seroprevalence of neutralizing antibodies to human adenovirus serotype 5 (Ad5) has been shown to range from 60 to 70%^23^. The elderly are more likely to have an exposure history to Ad5, with higher baseline neutralizing antibodies, decreasing the effectiveness of this vaccine strategy in the most vulnerable of populations. Such concerns were proven true in a phase II clinical trial, wherein individuals with pre-existing Ad5 vector immunity had a lower instance of seroconversion after the prime immunization^24^. Knowledge of this issue has led to utilizing low-seroprevalence adenovirus vectors like serotype 26 (Ad26) of the Janssen vaccine. These alternative low-seroprevalence vectors are even less immunogenic, and likely also suffer from pre-existing adenovirus T cells^23^. Further strategies to combat these issues included the investigation of a heterologous prime-boost regimen that would complicate vaccine production and rollout^25^.

In our study, no significant differences were seen between immune responses in mice with pre-existing immunity to RABV compared to naive mice. All vaccine groups had high antibody titers against RABV-G, S1, and the RBD and high neutralizing titers against SARS-CoV-2. Of note, RABV titers do not get increasingly boosted following subsequent immunizations. It has not been previously demonstrated that foreign antigens inserted into the RABV vaccine vector can continuously boost immune responses. However, in our experiments, while RABV-G titers level off following multiple immunizations, the S1 responses increase rapidly in the primary response and are greatly boosted in the secondary response.

Coronavirus immunity has been shown to wane rapidly^18^, because of which, the ability of a vaccine to elicit a prolonged immune response is important for long-term control of COVID-19. The RABV vaccine tends to elicit life-long immunity, but whether or not the RABV-vector system can confer the long-term responses to S1 of CORAVAX had not been demonstrated. Due to the lethality of rabies disease, current RABV pre-exposure prophylaxis requires the primary vaccine regimen followed by serological testing for protective titers every 6 months^26^. Although there is an initial decline in antibody titers post-vaccination, RABV titers have been shown to plateau and not decline much 3–7 years later^27^. Additionally, neutralizing titers have been shown to be detectable for up to 14 years^20^. There is evidence that it is the RABV-vector strategy that confers its long-term immune response to the foreign antigen. We have published on the longevity of the RABV-vector system in non-human primates with a tetravalent hemorrhagic fever vaccine^28^, and have additional supportive evidence in mice (unpublished).

The demand for a long-term COVID-19 vaccine is ever-increasing due to the continued mutation of SARS-CoV-2 and the emergence of variants of concern. At 4 months post-vaccination with the Pfizer mRNA vaccine BNT162b2, data showed titers at just 6% of peak levels^29^, and another study observed the same decline over 6 months^30^. In mice at 1-year post-vaccination, CORAVAX maintained 27% of peak S1 and 60% of RBD EC50 titers (Fig. 4c). Neutralizing antibodies have been shown to decline 6 months after the second dose, as short-lived plasmablasts may not develop into long-lived plasma cells; however, this switch to long-lived cells is observed after natural infection^31,32^. CORAVAX-immunized mice have highly significant numbers of long-lived cells specific to S1 and RBD in the bone marrow.

To the best of our knowledge, no other COVID-19 vaccines have demonstrated this potential for long-term immunity. We do not have mechanistic evidence for the longevity of the RABV vaccine platform, but we hypothesize that it is due to the stability of the RABV antigen. It may also be the case that foreign antigens are more stable in the RABV vector, or the cell targeting of the RABV antigen contributes to immune longevity. This is not something we have directly demonstrated, but an area of interest in the future. Further experiments will aim to demonstrate the presence of memory B cells able to respond rapidly to S1 in CORAVAX-immunized mice. We will also be testing the ability of CORAVAX to boost the immune response after an extended time post-initial vaccination.

All studies presented here aim at a solution for a safe, effective, and long-term COVID-19 vaccine with the potential for global distribution. The use of vaccine adjuvants generally allows for a lower required antigen dose for the same effect, decreasing the production demands of the vaccine itself. The inclusion of an affordable and highly effective adjuvant like MPLA-AddaVax in the formulation will decrease the cost per dose of CORAVAX. A vaccine that maintains high long-term responses, especially for a pathogen known to have waning immunity, would decrease and potentially eliminate the need for booster vaccinations. This is particularly important in the developing world where primary vaccinations of civilians are a challenge in itself. These areas are also limited by the requirement of vaccine cold-chain storage, but our RABV-vector platform is heat-stable and retains its immunogenicity after even 2 weeks at 50 °C^33^. Altogether, we believe these studies elucidate the advantages of the RABV-vectored vaccine platform as an effective strategy against SARS-CoV-2.

## Methods

### Animal ethics statement

This study was carried out in strict adherence to recommendations described in the Guide for the Care and Use of Laboratory Animals, the Office of Animal Welfare, and the United States Department of Agriculture. All animal work was approved by the Institutional Animal Care and Use Committee (IACUC) at Thomas Jefferson University. All procedures were carried out under isoflurane anesthesia by trained personnel and under the supervision of veterinary staff. Mice were housed in cages in groups of five, under controlled humidity, and temperature conditions, and 12-hour (h) light and dark cycles. Food and water were available ad libidum.

### Vaccines

The recombinant rabies vaccines BNSP333, FILORAB1, and CORAVAX were constructed, recovered, purified with sucrose, inactivated with β-propiolactone (BPL), and characterized. Briefly, the virus was used to inoculate Vero cells seeded in Cellstack Culture Chambers (Corning) and propagated in VP-SFM medium (Thermo Fisher Scientific) over a period of 18 days. Supernatant collected on day 10 post-infection was filtered through 0.45 µm PES membrane filters (Nalgene) and layered onto 20% sucrose in phosphate-buffered saline (PBS). Virions were sedimented by ultracentrifugation in an SW32 rotor for 1.5 h at 25,000 rpm. Viral particles were resuspended in PBS and inactivated with 50 μL per 1 mg of particles in a 1:100 dilution of BPL (Millipore Sigma, Cat# P5648) in cold water. The absence of infectious particles was verified by inoculating BSR cells with 10 μg of BPL-inactivated viruses over three passages. The vaccines were stored at −80 °C before use.

### Immunizations

Groups of five 6–8 week old C57BL/6 or BALB/c mice were purchased from Charles River Laboratories. As per the adjuvant study, 5 µg MPLAs in 2.5% AddaVax (Invitrogen), and 100 µg Adju-Phos (alum, Invivogen) were formulated with the immunization. In all subsequent experiments, mouse immunizations were adjuvanted with MPLA-AddaVax. Mice were inoculated intramuscularly with 10 µg of chemically inactivated CORAVAX particles in 100 µL of PBS and boosted with the same amount of virus 4 weeks later. For pre-existing immunity experiments, 4 weeks before CORAVAX immunization, mice were immunized with either 10 µg of chemically inactivated BNSP333 or FILORAB1 in 100 µL of PBS. Those mice and an additional group of naive mice were immunized 4 weeks later with CORAVAX as described above and boosted 4 weeks later. Blood samples were collected by retro-orbital bleed before the first immunization until the study endpoint.

### Antigen production for ELISA and ELISpot

For the production of HA-tagged S1 and His-tagged RBD glycoproteins, sub-confluent T175 flasks of 293 T cells (human kidney cell line) were transfected with a eukaryotic expression vector (pDisplay) that expresses HA-tagged glycoproteins, and purified over a column. Fractions were collected and analyzed by Western Blot with polyclonal antiserum against the S1 domain (Thermo Fisher, Cat# PA581798) or a mouse monoclonal RBD-specific antibody (InvivoGen, Cat# srbd-mab10). Peak fractions were then pooled and dialyzed against PBS in 10,000 molecular weight cutoff dialysis cassettes (Thermo Fisher Scientific) to remove excess HA peptide. After dialysis, the protein was quantitated by UV spectrophotometry and frozen in small aliquots at −80 °C. Stripped RABV Glycoprotein (G) antigen was produced as discussed preciously by infecting BEAS-2B cells with rVSV-ΔG-RABV-G-GFP in OptiPRO SFM. Viral supernatants were concentrated and ultracentrifuged through a 20% sucrose cushion. Viral pellets were then resuspended in detergent-containing buffer and centrifuged to strip antigen from the virus. All antigens were further analyzed and characterized by SDS-PAGE and Western Blot.

### SARS-CoV-2 anti-S1, anti-RBD, and anti-RABV-G IgG ELISA

Immulon 4 HBX 96-well flat-bottom Microtiter plates were coated overnight at 4 °C with 50 ng/well of recombinant S1, RBD, or RABV-G diluted in 15 mM Na_2_CO_3_, 35 mM NaHCO_3_ coating buffer. The plates were washed three times with 300 μL of PBS containing 0.05% Tween-20 (PBST), as were all succeeding washes, and then blocked for 2 h at room temperature (RT) in 5% Milk in PBST. The plates were washed and mouse sera were added at a 1:50 starting dilution and further diluted threefold down the plates. Plates were kept at 4 °C overnight, washed, and incubated for 2 h at RT with 100 μL per well horseradish peroxidase-conjugated goat anti-mouse IgG-Fc, IgG2c, IgG2a, or IgG1 antibody diluted in PBST 1:8000 for S1 and RBD, and 1:20,000 for RABV-G. The plates were then washed and developed by the addition of 200 μL per well of o-Phenylenediamine Dihydrochloride substrate. The reaction was stopped after 15 min by adding 50 μL per well of 3 M sulfuric acid. The plates were read at the absorbance wavelength of 630 nm (background) and 490 nm (experimental) on a BioTek ELx800 Plate Reader with GEN5 software. The 630 nm reading was subtracted from the 490 nm reading to calculate the delta value analyzed in GraphPad Prism 9 software.

### Rabies virus neutralization by RFFIT

Rapid fluorescent focus inhibition tests (RFFIT) were performed beginning with mouse neuroblastoma cells (NA) cultured in serum-enriched RPMI media, seeded in 96-well plates, and incubated for 48 h. Independently, individual mouse serum was serially diluted three-fold with a starting dilution range of 1:10 to 1:400, depending on the time point of the sera collection. A pre-diluted mixture of RABV strain CVS-11, previously determined to achieve 90% infection in confluent NA cells, was added to each serum dilution. Along with the sera dilutions, the US standard rabies immune globulin (WHO STD) at a starting dilution of 2 international units (IU) per mL was incubated with the virus mixture for 1 h at 34 °C. The medium was then removed from the NA cell plate, replaced with the sera/virus mix, and incubated for 2 h at 34 °C. Post infection, the sera/virus mixture was aspirated and replaced with a fresh medium. The plates were then incubated for 22 h (24-h total infection) at 34 °C. After incubation, cells were fixed with 80% acetone, dried, and stained with FITC anti-RABV N Monoclonal Globulin overnight at 34 °C. Wells were assessed for percent infection using a fluorescent microscope. The Reed-Muench method was used to calculate 50% endpoint titers; these were converted to IU/mL by comparing them to that of the WHO STD.

### SARS-CoV-2 neutralization assay

Neutralizing titers for SARS-CoV-2 were determined as discussed previously^4^. Briefly, serum was heat inactivated for 30 min at 56 °C, diluted 10-fold, and then further diluted in a 2-fold serial dilution in 96-well round-bottom plates in Opti-MEM (1% Penicillin-Streptomycin). Diluted serum was mixed with 100 PFU of SARS-CoV-2 and incubated for 1 h at 37 °C. 100 μL of the serum/virus mixtures were then transferred to Vero E6 cell monolayers in flat-bottom 96-well plates and incubated for 2 days at 37 °C. Cells were blocked with 2% BSA in PBST for 60 min and incubated in primary antibody (anti-dsRNA J2) at 4 °C overnight. Cells were washed three times with PBS and incubated in secondary (goat anti-mouse Alexa 488-ThermoFisher, Cat# A32723, and hoescht 33342-ThermoFisher, Cat# H3570) for 2 h at RT. Virus fluorescence was measured with a Cytation Hybrid Multi-Mode reader at 488 nm (Biotek Instruments) and the dilution displaying 100% neutralization was reported.

### Enzyme-linked immunosorbant spot (ELISpot) assay

An ELISpot assay was used to quantify the number of S1, RBD, and RABV-G-specific ASCs in mouse spleen and bone marrow samples. ELISpot plates were coated with S1 (10 μg/mL), RBD (20 μg/mL), and RABV-G (10 μg/mL) in PBS overnight at 4 °C. Plates were washed with PBS and blocked with goat serum for 1 h at 37 °C followed by 4 °C during the preparation of cells. Bone marrow was harvested from the mouse femurs and tibias. These and the spleens were homogenized followed by red blood cell lysis in ACK buffer; 1.5 × 10^6^ cells were added to antigen-coated plates, serially diluted, and incubated at 37 °C for 16 h. Plates were washed with PBST and incubated in HRP-conjugated goat anti-mouse IgG-Fc, IgG2a, or IgG1 in PBST for 1–2 h at 37 °C. Plates were washed with PBST, followed by PBS, and TrueBlue peroxidase substrate was added. The reaction was quenched with water and plates were counted on an AID ELISpot reader.

### Statistical analysis

For all mouse experiments, a minimium of five mice per group were used to obtain statistical power as recommended by a biostatistician. For the ELISA, log-transformed half-maximal effective concentration (EC50) titers were determined by a 3-fold dilution series of delta OD value (OD 490–630 nm). For all statistical analyses, one-way ANOVA with post-hoc Tukey’s test was performed on log-transformed data for each time point. *p* > 0.1234 (ns), *p* < 0.0332 (*), *p* < 0.0021 (**), *p* < 0.0002 (***), *p* < 0.0001 (****).

### Reporting summary

Further information on research design is available in the Nature Research Reporting Summary linked to this article.

## Supplementary information

Supplementary Information

REPORTING SUMMARY

## Data Availability

All relevant data are available from the corresponding authors upon request.

## References

[1] Coronavirus Resourse Center https://coronavirus.jhu.edu (2022).

[2] Landscape of candidate vaccines in clinical development https://www.who.int/publications/m/item/draft-landscape-of-covid-19-candidate-vaccines (2022).

[3] Kurup D, Wirblich C, Ramage H, Schnell MJ (2020). Rabies virus-based COVID-19 vaccine CORAVAX™ induces high levels of neutralizing antibodies against SARS-CoV-2. NPJ Vaccines.

[4] Kurup D (2021). Inactivated rabies virus vectored SARS-CoV-2 vaccine prevents disease in a Syrian hamster model. PLOS Pathog..

[5] Rupprecht CE, Nagarajan T, Ertl H (2016). Current status and development of vaccines and other biologics for human rabies prevention. Expert Rev. Vaccines.

[6] Abreu-Mota T (2018). Non-neutralizing antibodies elicited by recombinant Lassa-Rabies vaccine are critical for protection against Lassa fever. Nat. Commun..

[7] Blaney JE (2011). Inactivated or live-attenuated bivalent vaccines that confer protection against rabies and Ebola viruses. J. Virol..

[8] Keshwara R (2019). A recombinant rabies virus expressing the marburg virus glycoprotein is dependent upon antibody-mediated cellular cytotoxicity for protection against marburg virus disease in a murine model. J. Virol..

[9] Keshwara R (2019). Rabies-based vaccine induces potent immune responses against Nipah virus. NPJ Vaccines.

[10] Kurup D, Wirblich C, Feldmann H, Marzi A, Schnell MJ (2015). Rhabdovirus-based vaccine platforms against henipaviruses. J. Virol..

[11] Faber M (2005). A single amino acid change in rabies virus glycoprotein increases virus spread and enhances virus pathogenicity. J. Virol..

[12] Scher G, Schnell MJ (2020). Rhabdoviruses as vectors for vaccines and therapeutics. Curr. Opin. Virol..

[13] Johnson RF (2016). An inactivated rabies virus-based ebola vaccine, FILORAB1, adjuvanted with glucopyranosyl lipid A in stable emulsion confers complete protection in nonhuman primate challenge models. J. Infect. Dis..

[14] Rabies https://www.who.int/health-topics/rabies (2022).

[15] A Look at Each Vaccine: Rabies Vaccine. https://www.chop.edu/centers-programs/vaccine-education-center/vaccine-details/rabies-vaccine (2022).

[16] Hampson K (2015). Estimating the global burden of endemic canine rabies. PLoS Negl. Trop. Dis..

[17] The Weekly Epidemiological Record. (ed. WHO/Department of Control of Neglected Tropical Diseases) **92**, 77–86 (2017).

[18] Edridge AWD (2020). Seasonal coronavirus protective immunity is short-lasting. Nat. Med..

[19] Zheutlin, A. et al. Durability of protection against COVID-19 breakthrough infections and severe disease by vaccines in the United States. *medRxiv*10.1101/2022.01.05.22268648 (2022).

[20] Strady A (1998). Antibody persistence following preexposure regimens of cell-culture rabies vaccines: 10-year follow-up and proposal for a new booster policy. J. Infect. Dis..

[21] A Phase 1, Open Label, Dose Escalation, Randomized, Multicenter Study to Evaluate the Reactogenicity, Safety, and Immunogenicity of an Intramuscular Inactivated Rabies Vector Platform Corona Virus Vaccine (rDNA-BBV151) in Healthy Volunteers., http://ctri.nic.in/Clinicaltrials/showallp.php?mid1=58694&EncHid=&userName=BBV151 (2021).

[22] Reed SG, Orr MT, Fox CB (2013). Key roles of adjuvants in modern vaccines. Nat. Med..

[23] Fausther-Bovendo H, Kobinger GP (2014). Pre-existing immunity against Ad vectors: humoral, cellular, and innate response, what’s important?. Hum. Vaccin Immunother..

[24] Zhu F-C (2020). Immunogenicity and safety of a recombinant adenovirus type-5-vectored COVID-19 vaccine in healthy adults aged 18 years or older: a randomised, double-blind, placebo-controlled, phase 2 trial. Lancet.

[25] Liu X (2021). Safety and immunogenicity of heterologous versus homologous prime-boost schedules with an adenoviral vectored and mRNA COVID-19 vaccine (Com-COV): a single-blind, randomised, non-inferiority trial. Lancet.

[26] Preexposure Vaccinations https://www.cdc.gov/rabies/specific_groups/travelers/pre-exposure_vaccinations.html.

[27] Mansfield KL (2016). Rabies pre-exposure prophylaxis elicits long-lasting immunity in humans. Vaccine.

[28] Kurup D (2021). Tetravalent rabies-vectored filovirus and lassa fever vaccine induces long-term immunity in nonhuman primates. J. Infect. Dis..

[29] Khoury J (2021). COVID-19 vaccine – Long term immune decline and breakthrough infections. Vaccine.

[30] Naaber P (2021). Dynamics of antibody response to BNT162b2 vaccine after six months: a longitudinal prospective study. Lancet Reg. Health Eur..

[31] Mistry, P. et al. SARS-CoV-2 variants, vaccines, and host immunity. *Front. Immunol.* **12**, 809244 (2022). 10.3389/fimmu.2021.809244 PMC8761766 35046961

[32] Turner JS (2021). SARS-CoV-2 infection induces long-lived bone marrow plasma cells in humans. Nature.

[33] Kurup D (2019). Inactivated rabies virus-based ebola vaccine preserved by vaporization is heat-stable and immunogenic against ebola and protects against rabies challenge. J. Infect. Dis..

