# Supplementary material for: Inactivated rabies-vectored SARS-CoV-2 vaccine provides long-term immune response unaffected by vector immunity
Source: NPJ Vaccines. 2022 Sep 23;7:110. doi: 10.1038/s41541-022-00532-7 (PMC9508099; doi:10.1038/s41541-022-00532-7)
Supplement: Supplementary file 1 — Supplementary Information [file 41541_2022_532_MOESM1_ESM.pdf]

Supplementary Figure 1

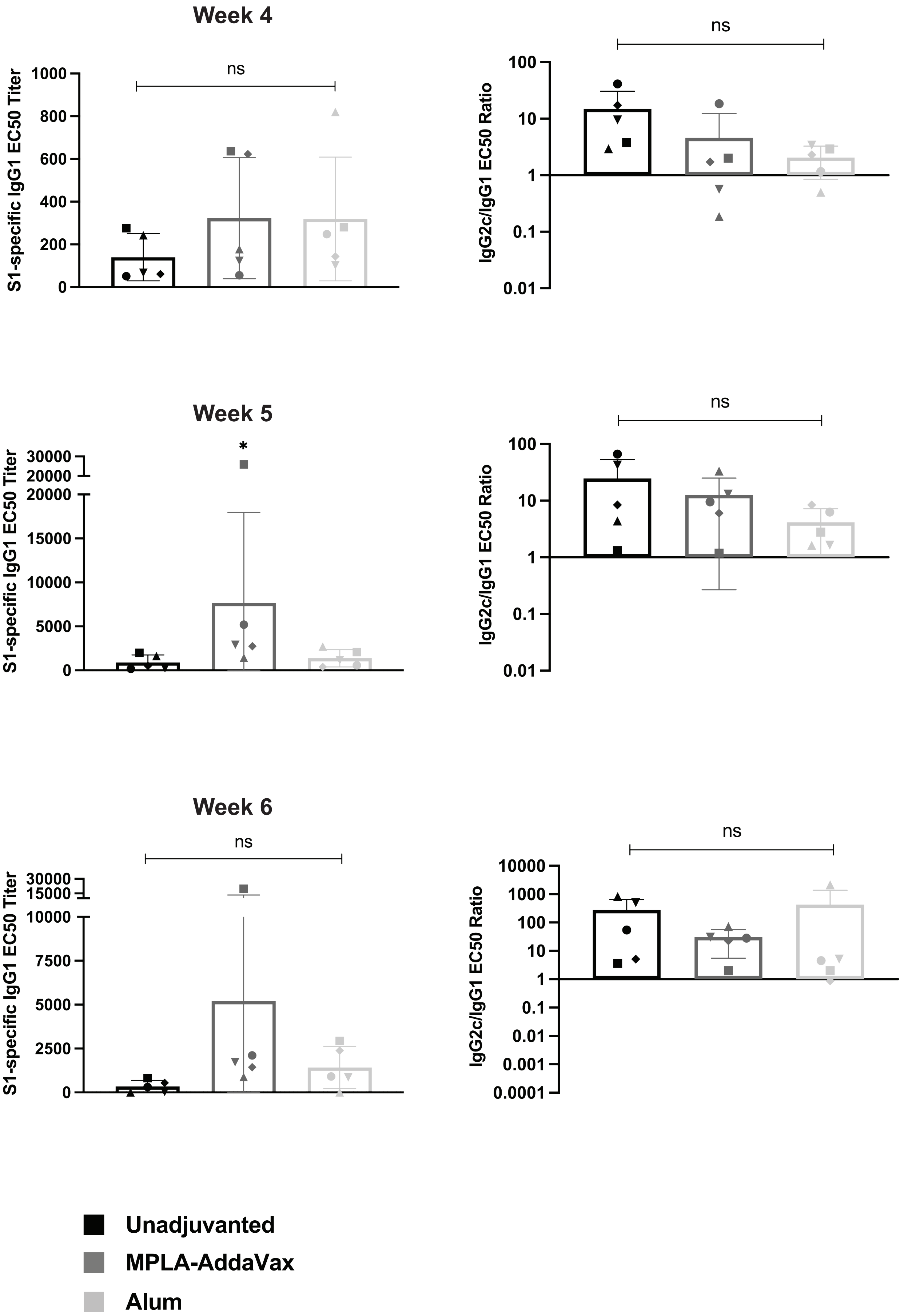

Supplementary Figure 1. S1-specific IgG1 responses and isotype subclass ratio of adjuvant comparison. C57BL/6 mice (n=5 per group, female) were immunized with 10 μg of CORAVAX without adjuvant, with MLPA-AddaVax, or with alum at day 0 and week 4. S1-specific IgG1 ELISA at weeks 4, 5, and 8 post-prime immunization reported as average half-maximal effective concentration (EC50) titer (bars) determined from individual mouse serum (symbols) ELISA curves. The average ratio of IgG2c/IgG1 EC50 titers titers (bars) determined from individual mouse (symbols) ELISA curves at weeks 4, 5, and 8. Error bars represent SD from the mean. Statistics are by one-way ANOVA with post-hoc Tukey's test of log-transformed EC50 titers. $p > 0.1234$ (ns), $p < 0.0332$ (*), $p < 0.0021$ (**), $p < 0.0002$ (***), $p < 0.0001$ (****).

Supplementary Figure 2

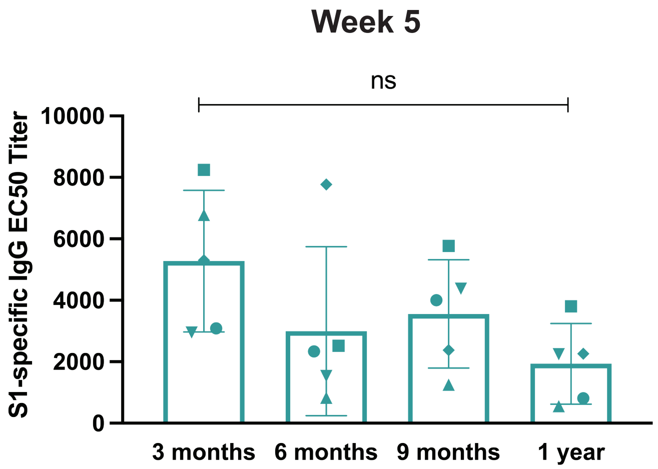

Supplementary Figure 2. CORAVAX immunization results in similar S1 titers at week 5 for long-term experiment groups. BALB/c mice (n=5 per group, female) were analyzed for S1-specific IgG by ELISA of immunized mouse sera one-week post-boost immunization (week 5). Average EC50 titers (bars) were determined from individual mouse (symbols) ELISA curves. Error bars represent SD from the mean. Statistics are by one-way ANOVA with post-hoc Tukey's test of log-transformed EC50 titer. $p > 0.1234$ (ns), $p < 0.0332$ (*), $p < 0.0021$ (**), $p < 0.0002$ (***), $p < 0.0001$ (****).
